# Gα_13_ Stimulates the Tyrosine Phosphorylation of Ric-8A

**DOI:** 10.5334/1750-2187-10-3

**Published:** 2015-07-27

**Authors:** Mingda Yan, Ji Hee Ha, Danny N. Dhanasekaran

**Affiliations:** Stephenson Cancer Center and Department of Cell Biology, 975 NE 10th Street, The University of Oklahoma Health Sciences Center, OK 73104, United States

**Keywords:** Gα_13_, Ric-8A, membrane-translocation, Src, Cdc42, p38MAPK

## Abstract

The G12 family of heterotrimeric G proteins is defined by their α-subunits, Gα_12_ and Gα_13_. These α-subunits regulate cellular homeostasis, cell migration, and oncogenesis in a context-specific manner primarily through their interactions with distinct proteins partners that include diverse effector molecules and scaffold proteins. With a focus on identifying any other novel regulatory protein(s) that can directly interact with Gα_13_, we subjected Gα_13_ to tandem affinity purification-coupled mass spectrometric analysis. Our results from such analysis indicate that Gα_13_ potently interacts with mammalian Ric-8A. Our mass spectrometric analysis data also indicates that Ric-8A, which was tandem affinity purified along with Gα_13_, is phosphorylated at Ser-436, Thr-441, Thr-443 and Tyr-435. Using a serial deletion approach, we have defined that the C-terminus of Gα_13_ containing the guanine-ring interaction site is essential and sufficient for its interaction with Ric-8A. Evaluation of Gα_13_-specific signaling pathways in SKOV3 or HeyA8 ovarian cancer cell lines indicate that Ric-8A potentiates Gα_13_-mediated activation of RhoA, Cdc42, and the downstream p38MAPK. We also establish that the tyrosine phosphorylation of Ric-8A, thus far unidentified, is potently stimulated by Gα_13_. Our results also indicate that the stimulation of tyrosine-phosphorylation of Ric-8A by Gα_13_ is partially sensitive to inhibitors of Src-family of kinases, namely PP2 and SI. Furthermore, we demonstrate that Gα_13_ promotes the translocation of Ric-8A to plasma membrane and this translocation is attenuated by the Src-inhibitors, SI1 and PP2. Thus, our results demonstrate for the first time that Gα_13_ stimulates the tyrosine phosphorylation of Ric-8A and Gα_13_-mediated tyrosine-phosphorylation plays a critical role in the translocation of Ric-8A to plasma membrane.

## Introduction

The G proteins, Gα_12_ and Gα_13_ are defined by their a subunits and are critically associated with signaling pathways mediating cell proliferation, differentiation, and neoplastic transformation [1]. It has also been shown that the signaling pathways regulated by G proteins, including those of Gα_12_ and Gα_13_, are spatially and temporally regulated by proteins that can directly interact with the α-subunits of the respective G proteins and/or their cognate upstream receptors [2]. The proteins that primarily interact with Gα_12_ and Gα_13_ include the Rho-GEF family of proteins [3], Btk/Tec family nonreceptor tyrosine kinases, GAP1-subfamily of Ras-GAP proteins [4], Ser/Thr protein phosphatase type 5 [5], cadherins [6], actin binding protein radixin [7], cortactin-binding protein Hax-1 [8] and scaffold protein JLP [9]. In this context, it is worth noting that tandem affinity purification couple mass spectrometric analysis has been used to identify novel protein-protein interactions. However, such an approach has been utilized to identify Gα-interacting proteins. Therefore, with a focus on identifying any novel regulators of Gα_13_, we carried out a tandem affinity purification (TAP) coupled mass spectrometric analysis with Gα_13_. Our results identified mammalian Ric-8A protein as one of the α-subunit interaction protein of Gα_13_.

RIC-8A, which is also known as synembryn, was originally identified as an acetylcholinesterase inhibitor, in a genetic screen for resistance to aldicarb, in *C. elegans* [10]. Initial studies with *C. elegans* indicated that RIC8A is upstream of Gα_o_-Gα_q_ signaling network that regulates synaptic transmission [10] and is also upstream of Gα_o_-mediated signaling involved in the asymmetric cell division of *C. elegans* embryos [11,12]. Subsequently, two distinct mammalian *RIC8A* homologues, *RIC8A* and *RIC8B genes* that encode Ric-8A and Ric-8B, were identified by yeast two-hybrid screens using Gα_o_ and Gα_s_ as baits [13]. *In vitro* interaction studies have shown that Ric-8A also interacts with Gα_q_, Gα_i_, and albeit weakly, with Gα_13_ [13]. These studies have also demonstrated that Ric-8A functions as a novel regulatory protein that promotes guanine nucleotide exchange in the α-subunits, primarily through the stabilization of the nucleotide-free transition state of the α-subunits. It has also been shown that Ric-8A protein stimulates the release of GDP from the α-subunit so that GTP-loading can occur in the guanine nucleotide-free α-subunits [13]. However, the unequivocal role of Ric-8A in its interaction with Gα_13_ and the functional significance of such an interaction remain to be clarified.

In addition to identifying Ric-8A as one of the major Gα_13_-interacting proteins, we also establish the endogenous interaction between Gα_13_ and Ric-8A in ovarian cancer cells. Furthermore, our TAP-couple mass spectrometric analysis indicates that Ric-8A is phosphorylated at Ser-436, Thr-441, Thr-443 and Tyr-435. Our studies also demonstrate that Ric-8A potentiates Gα_13_-mediated activation of the small GTPases RhoA, Cdc42 and the downstream p38MAPK. More interestingly, our results for the first time indicates that Gα_13_ promotes the tyrosine phosphorylation of Ric-8A through a mechanism involving Src-family of kinases, and this phosphorylation of tyrosine is a necessary event for Gα_13_-stimulated translocation of Ric-8A, to the plasma membrane.

## Experimental Procedures

### Cell culture and treatment

Human embryonic kidney cells (HEK293), ovarian cancer cells (SKOV3, HeyA8, and SKOV3ip) and African green monkey fibroblast cells (COS7) were maintained in Dulbecco modified Eagle medium (DMEM) containing 10% fetal bovine serum and 1% penicillin/streptomycin (Mediatech, Manassas, VA) at 37°C, in a 5% CO_2_ incubator. Transfection of HEK293 and COS7 cells was carried out using FuGENE 6 reagent (Roche, Indianapolis, IN) with a Reagent: DNA ratio of 3:1. Transfection of SKOV3 and HeyA8 cells were carried out using an Amaxa Nucleofector II system (Lonza, Walkersville, MD) using the manufacture’s protocol for the respective cell types. 24 hours after transfection the cells were harvested or utilized for further treatment studies. For the Src inhibitor experiments, the DMSO vehicle control or 10 µM of the inhibitors Src inhibitor 1 or PP2 (Calbiochem, San Diego, CA), were added 6 hours after transfection, and were treated/incubated overnight.

### DNA constructs

The amino acid sequence of N-terminal FLAG-tag-Strep-tag II (FS) was based on previously published procedures [14]. pcDNA3.1/Zeo(+) vector (Invitrogen, Carlsbad, CA) expressing the N-terminal 44 amino acid peptide with FLAG and Strep-(x 2) tags was constructed by overlapping PCR method. In brief, the strands of complementary oligonucleotides encoding the N-terminal 44 amino acids of the Flag tag (DYKDDDDK), followed by two Strep-tags (WSHPQFEK), were amplified by PCR. The following overlapping oligonucleotides designed by DNAWorks (http://helixweb.nih.gov/dnaworks/) with engineered *Nhe*I and *Afl*II (underlined) sites were used.

*5’GATCGCTAGCATGGACTATAAGGATGACGATGATAAGGGGAGCGCCGCA3’*
*5’AGCCTCCGCCTTTCTCGAACTGGGGATGGCTCCAGCTTGCGGCGCTCCCC
3’*
*5’CGAGAAAGGCGGAGGCTCCGGGGGAGGCAGCGGAGGAGGGTCCTGGTCCC
3’*
*5’GATCCTTAAGATATCGATAGCGCCTTTTTCGAATTGAGGATGGGACCAGGACCCTCCT
3’*

The PCR product encoding the tags was cloned into the pcDNA3.1/Zeo(+) vector, at *Nhe*I and *Afl*II sites, and the sequence was verified by DNA-sequencing. The resultant pcDNA-FS vector was then used to shuttle Gα_13,_ as well as the deletion mutants of Gα_13_. The open reading frame of human Gα_13_ was amplified by PCR from pCMV-SPORT6-Gα_13_ (Open BioSystems, Huntsville, AL) using the following primers with engineered *Cla*I and *Eco*RI sites (underlined):

*5’
CTGAATCGATGCGGACTTCCTGCCGTCGCGGT
3’*
*5’
TTTGGAATTCACTGTAGCATAAGCTGCTTGAGGT
3’*

The Gα_13_-insert thus obtained, was cloned in to the FS vector to fuse with N-terminal TAP tag. Gα_13_QL (Q226L) mutation was generated by QuikChange method using the following two site-directed mutagenesis (underlined) oligonucleotides:

*5’CCTTTCAAAATGGTTGATGTAGGaGGcCtGAGATCAGAAAGGAAACGTTGG3’*
*5’CCAACGTTTCCTTTCTGATCTCAGGCCTCCTACATCAACCATTTTGAAAGG3’*

Deletion constructs of Gα_13_ were generated using the following primer pairs with engineered restriction sites (underlined):

ΔC: aa 1–340 (*Cla*I and *Bam*HI)

*5’CTGAATCGATGCGGACTTCCTGCCGTCGCGGT
3’*
*5’TGGTGAATTCACTTCTGTTGCTGGTCCCGGCGTT3’*

N: aa 1–226 (*Cla*I and *Bam*HI)

*5’CTGAATCGATGCGGACTTCCTGCCGTCGCGGT3’*
*5’CGGGATCCGTCTGACCACCTACATCAACC3’*

C: aa 224–377 (*Eco*RI and *Kpn*I)

*5’CGGAATTCTGGTGGTCAGAGATCAGAAAGG3’*
*5’GGGGTACCTCACTGTAGCATAAGCTGCTTG3’*

D4: aa 259–377 (*Cla*I and *Eco*RI)

*5’
TATG*atcGAT*CGACTGACCAATCGCCTTAC
3’*
*5’
TTTG*GAATTC*ACTGTAGCATAAGCTGCTTGAGGT3’*

D5: aa 302–377 (*Cla*I and *Eco*RI)

*5’GAAGATCGATATTGTGAGCATCAAAGACTATTTCCTAG3’*
*5’TTTGGAATTCACTGTAGCATAAGCTGCTTGAGGT3’*

D6: 342–377 (*Cla*I and *Eco*RI)

*5’ACAGATCGATTTATACCACCACTTCACCACTGCTATC3’*
*5’TTTGGAATTCACTGTAGCATAAGCTGCTTGAGGT3’*

D8: 302–340 (*Cla*I and *Eco*RI)

*5’GAAGATCGATATTGTGAGCATCAAAGACTATTTCCTAG3’*
*5’TGGTGAATTCACTTCTGTTGCTGGTCCCGGCGTT3’*

PCR products were inserted into the pcDNA3-FS vector using the appropriate restriction site. In the case of construct C, the insert was first placed into a shuttling vector pECFP-C1 (Clontech, Mountain View, CA), following which it was subcloned into the FS vector by *Xho*I and *Apa*I. Vector encoding Ric-8A (pCMV-SPORT6-Ric-8A) was purchased from Open BioSystems. This construct was used to generate N-terminal HA- or GFP-tagged Ric-8A by Gateway cloning system. Ric-8A engineered with attB1 and attB2 sites (underlined) was amplified from pCMV-SPORT6-Ric-8A using following primers, and cloned in pDONR221 donor vector by Gateway BP clonase.

*5’GGGGACAAGTTTGTACAAAAAAGCAGGCTCCATGGAGCCCCGGGCGGTTGCAGAA3’*
*5’GGGGACCACTTTGTACAAGAAAGCTGGGTCAGTCAGGGTCCGAGTCAGGGTCCGA3’*

The Ric-8A in the entry construct was further cloned in pcDNA3-HA or pcDNA3-GFP destination vector by Gateway LR clonase by inserting a gateway Reading Frame A (Invitrogen) in the *Eco*RV site, and a FS fragment between *Nhe*I and *Cla*I sites was replaced by HA fragment or GFP open reading frame without stop codon. The HA fragment was obtained by annealing the following complementary oligos (underlined: *Nhe*I and *Cla*I)

*5’AGCTGGCTAGCATGGCCTACCCTTATGACGTGCCAGATTATGCCATCGATTCGAC3’*
*5’GTCGAATCGATGGCATAATCTGGCACGTCATAAGGGTAGGCCATGCTAGCCAGCT3’*

The GFP open reading frame without stop codon was amplified by the following oligos (underlined: *Nhe*I and *Cla*I), using pCRE-d2EGFP (Clontech) as the template

*5’AGCAGctagCATGGTGAGCAAGGGCGAGGAGCT
3’*
*5’GAAGatcgaTCTTGTACAGCTCGTCCATGCCGAGAGT
3’*

### Protein Purification and Identification

HEK293 (2 × 10^6^ cells/dish, 8 dishes) cells were transfected with FS or FS-Gα_13_QL construct (6 mg) and propagated for 48 hours. Cells were rinsed with cold PBS and lysed by incubating for 30 minutes, in a lysis buffer containing 50 mM Tris-Cl, pH 7.4, 150 mM NaCl, 1 mM EDTA, 0.1% NP40, 5% Glycerol, and Sigma’s Protease Inhibitor Cocktail (1:200 dilution; Sigma P8340). The lysates were centrifuged and used for further experiments. For the first affinity purification, the cell lysate (13 mg) was bound with Strep-Tactin resin (IBA, Gottingen, Germany), washed by lysis buffer, and eluted by desthiobiotin. For the second affinity purification, anti-FLAG M2 resin (Sigma, Saint Louis, MO) was used for binding and 3 × FLAG peptide was used for elution. The purified protein complexes were separated by SDS-PAGE and visualized by SilverQuest silver staining (Invitrogen) or ProtoBlue Safe staining (National Diagnostics, Atlanta, GA). Protein identification in the band of interest carried out using MALDI-TOF mass spectrum (Protein Core Facility, Columbia University Medical Center, New York, NY).

### Immunoprecipitation

Immunoprecipitation analyses using specific antibodies were carried out using previously published methods [15]. Cells were rinsed by cold PBS and scraped in lysis buffer (as mentioned in the previous section). The cell lysates thus obtained, were quantified using Bradford method (Bio-Rad, Hercules, CA), and utilized for the immunoprecipitation experiments. For the immunoprecipitation with the FLAG antibody, 1 mg of the total protein lysate was incubated with 10 µl of anti-FLAG M2 EZview beads (Sigma) for 5 hours, at 4°C. The immune complexed beads were washed in lysis buffer (3×) and resuspended in Laemmli’s sample buffer and resolved using immunoblot analysis. For the endogenous protein immunoprecipitation, 1 mg of the total protein lysate was incubated with 1 µg of normal mouse-IgG, Gα_13_ (clone 73.1; Santa Cruz Biotechnology, Santa Cruz, CA) or Ric-8A-antibody (clone 3G3, OriGene, Rockville, MD) for 2 hours. This was followed by incubation with 10 µl of Protein G EZview beads for 16 hrs, at 4°C. The beads were washed three times with lysis buffer, resuspended in Laemmli’s sample buffer and resolved further.

### Immunoblot Analysis

Samples were separated by SDS-PAGE and electroblotted onto polyvinylidene difluoride membranes for immunoblot analysis, according to previously published methods [16]. The primary antibodies, Ric-8A (clone 3G3, OriGene), Gα_13_ (AS1–89–2 [8], Gα_12_ (S-20), Gα_q_ (E-17), Gα_i-2_ (L5), Cdc42 (P1), Rac1 (23A8), RhoA (119) (Santa Cruz Biotechnology), phospho-Serine, phospho-Threonine (Abcam, Cambridge, MA), phospho-Tyrosine and phospho-Cdc42/Rac1 (Ser-71) (Cell Signaling, Danvers, MA) were used for the immunoblot studies. Peroxidase-conjugated anti-rabbit IgG and anti-mouse IgG secondary antibodies were purchased from Promega (Madison, WI) and GE Healthcare (Waukesha, WI), respectively. Enhanced chemiluminescence was performed using Pierce substrates (Thermo Scientific, Rockford, IL) and imaged on a Kodak Image Station (Carestream Health, Rochester, NY). Band density was quantified by ImageJ software (National Institutes of Health) and the statistical significance was calculated with the unpaired Student’s *t*-test from Prism software (GraphPad, La Jolla, CA).

### Fluorescent microscopy

COS7 cells (1 × 10^6^ cell/dish) were transfected with pcDNA3-GFP or pcDNA3-GFP-Ric-8A together with empty vector or pcDNA3-Gα_13_WT constructs. 24 hours after transfection, the live cells were observed using a Nikon Eclipse TE2000-E fluorescent microscope with a 40x objective lens at aperture 0.9 and fluorescent images were captured by MetaMorph microscopy software (Molecular Devices, Sunnyvale, CA).

### RhoA, Rac1, and Cdc42 activation assay

Activated RhoA, Rac1, or Cdc42 was monitored using respective GST-fused binding domain pull-down assays as described previously [8]. A bacterial expression construct encoding glutathione S transferase (GST)-fused Rhotekin Rho-binding domain (RBD) or PAK3 p21-binding domain (PBD) was transformed in *E. coli* BL21DE3 strain and the IPTG-induced GST-fusion protein was purified further by using Glutathione Sepharose 4B beads (GE Healthcare). Cells were lysed in a magnesium lysis buffer (25 mM HEPES, pH 7.5, 150 mM NaCl, 10 mM MgCl_2_, 1 mM EDTA, 10% glycerol, 1% NP40, and protease inhibitor cocktail) the activated GTP-bound RhoA from clarified cell lysates (1 mg), was pulled down using 10 ml of GST-Rhotekin RBD suspension, and identified using Immuno blotting. Similarly, active GTP-bound Rac1 and Cdc42 were assayed by pulling down active Rac1 and Cdc42 with GST-PAK PBD beads (10 µl for 1 mg of lysate). Activated Rac1 versus Cdc42 were resolved by immunoblot analysis with anti-Rac1 and anti- Cdc42 antibodies, respectively.

### Statistical analysis

Statistical analysis was carried out with GraphPad Prism (GraphPad, La Jolla, CA) by a 2-tailed Student *t* test with Welch correction.

## Results

### Interaction of Ga13 with Ric-8A

**Figure 1:**
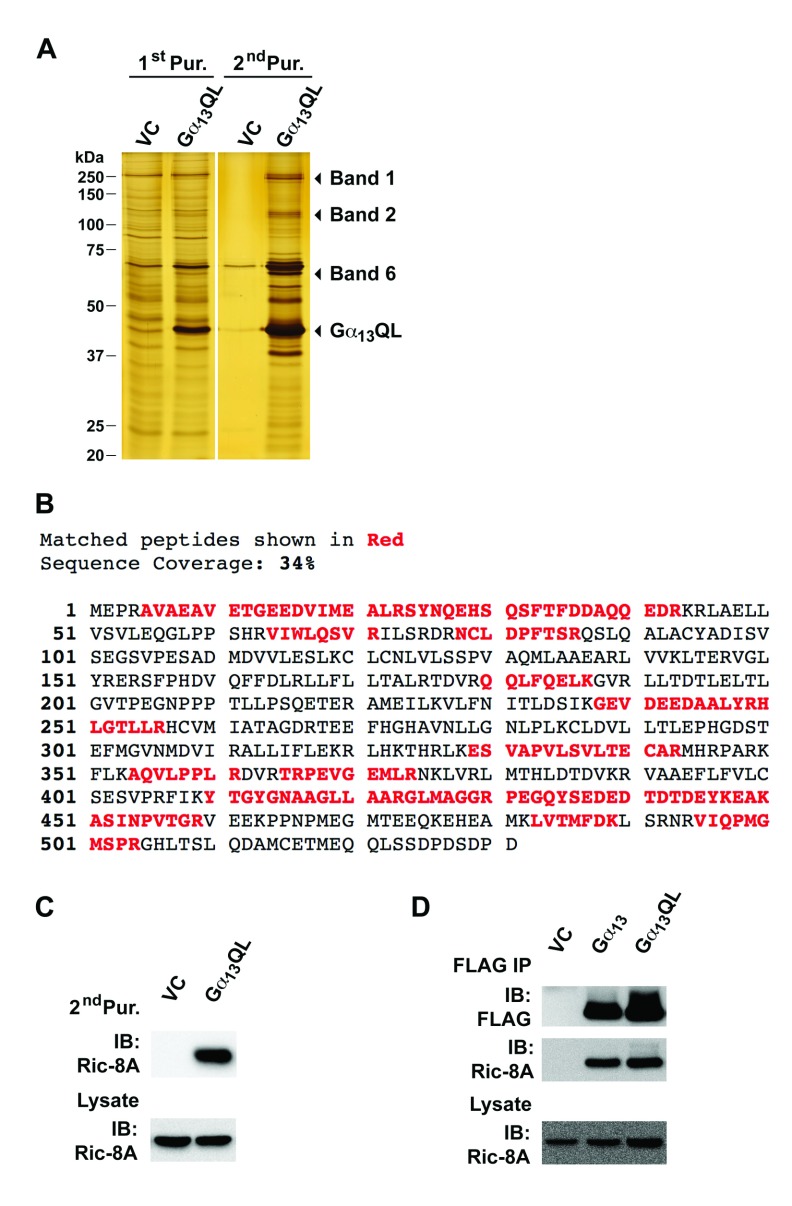
Identification of Ric-8A as Gα_13_-interacting protein. **A.** Silver staining profiles of tandem affinity purified proteins from HEK293 cells transfected with FS-tagged Gα_13_QL construct or vector control (VC). First affinity purification (1^st^ Pur.) was carried out using Strep-Tactin resin in which the proteins bound to the resin were eluted using desthiobiotin, resolved by SDS-PAGE electrophoresis and visualized by silver staining. Second affinity purification (2^nd^ Pur.) was carried out using anti-FLAG M2 resin in which the bound proteins were eluted using FLAG peptide. The eluted proteins were resolved by 10% SDS-PAGE and visualized by silver staining. **B.** Mass spectrometric analysis of band 6. Matched peptides (in red) cover 34% of human Ric-8A protein. **C.** Lysates from vector control and Gα_13_QL-tranfectants were processed through second affinity purification (2^nd^ Pur.). The bound proteins eluted by FLAG-peptides were resolved in by SDS-PAGE and subjected to immunoblot analysis using Ric-8A-antibodies. **D.** Lysates from HEK293 cells transfected with FS-tagged wild-type Gα_13_ (Gα_13_), activated mutant of Gα_13_ (Gα_13_QL), or FS-tag-vector control (VC), were immunoprecipitated with FLAG antibody and assessed for the presence of Ric-8A.

To identify novel Gα_13_-interacting proteins, a tagged, constitutively active mutant of Gα_13_ was engineered with Flag-Strep-epitope (pcDNA3-FS-Gα_13_Q226L) and subsequently expressed in HEK293 cells, along with vector control. Following the two-step purification, several major protein bands were observed by silver staining method (Figure 1A). Analyses of these selected protein bands using MALDI-TOF mass spectrometric analysis, identified two of the well-characterized Gα_13_-interacting proteins, namely, leukemia associated Rho guanine nucleotide exchange factor (Band 1: LARG) and p115 Rho guanine nucleotide exchange factor (Band 2: p115RhoGEF), thus validating our approach to identify Gα_13_-interacting proteins. More interestingly, the protein band denoted as Band 6 (Figure 1A), was identified as mammalian Ric-8A (Figure 1B). This identification was further substantiated by immunoblot analysis with anti-Ric-8A antibody (Figure 1C). Previous studies have shown that the Ric-8A binding to Gα_i1_ is independent of the activation status of Gα_i1_ [13,17]. Therefore we investigated whether the activation of Gα_13_ has any effect on its interaction with Ric-8A. To test, HEK293 cells were transiently transfected with FS-tagged wild-type Gα_13_ (Gα_13_), activated mutant of Gα_13_ (Gα_13_QL), or FS-tag-vector control (VC). At 48 hrs, the cells were lysed and the FLAG-tagged Gα_13_ or Gα_13_Q226L was immunoprecipitated with FLAG antibody and assessed for the presence of coimmunoprecipitaed Ric-8A by immunoblot analysis. The results indicated that Ric-8A, co-immunoprecipitated to an equal extent, with Gα_13_WT and Gα_13_Q226L, thereby indicating that the interaction between Ric-8A and Gα_13_ is regardless of the activation-status of Gα_13_ (Figure 1D). This is consistent with the proposed function of Ric-8A in which it interacts with the GDP-bound configuration of the Gα-subunits to promote GDP-dissociated nucleotide-free configuration of the α-subunit for GTP-loading [13,17,18]. Therefore, wild type Gα_13_ construct used in subsequent Ric-8A-Gα_13_ interaction studies.

### In Vivo Interactions of Ric-8A

**Figure 2:**
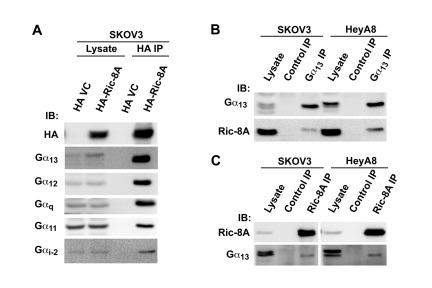
*In Vivo* interactions of Ric-8A. **A.** HA-epitope tagged Ric-8A (pcDNA3-HA-Ric-8A) along with the vector control was transiently expressed in SKOV3 cells (2 × 10^6^ cells) for 48 hrs. Ric-8A from the lysates was immunoprecipitated, using antibodies to HA-epitope. Endogenous Gα_13_, Gα_12_, Gα_q_, Gα_11_ or Gα_i2_ that interacted with ectopically expressed Ric-8A was monitored by coimmunoprecipitation, followed by immunoblot analysis with the respective antibodies. Data presented is a representation of three independent experiments. **B.** Lysates from SKOV3 or HeyA8 cells (2 × 10^6^ cells) were incubated with normal mouse IgG (Control IP) or anti- Gα_13_ antibody. Gα_13_-bound immunecomplex in the lysates was precipitated by protein-G resin and analyzed for the presence of endogenous Ric-8A by immunoblot analysis (IB). **C.** Lysates from SKOV3 or HeyA8 cells were incubated with normal mouse IgG (Control IP) or anti-Ric-8A antibody. The bound protein complex was precipitated by protein-G resin and analyzed for the presence of endogenous Gα_13_ by immunoblot analysis.

It should be noted here that previous studies have shown albeit a weaker interaction between Gα_13_ and GST-tagged Ric-8A [13,19]. While our studies have confirmed the interaction between RIC-8A and Gα_13_, we further sought to substantiate the presence of such an interaction *in vivo*. Since we have previously shown that the Gα_13_ plays a critical signaling role in ovarian cancer cells [20], we initiated the interaction studies between Gα_13_ and Ric-8A in these cells. HA-tagged Ric-8A was transiently expressed in the ovarian cancer cell line SKOV3 (2 × 10^6^ cells). At 48 hrs following transfection, lysates were prepared from these transfectants and Ric-8A from the lysates was immunoprecipitated using HA-epitope antibody. The immunoprecipitates were analyzed for the presence of Gα_13_ by immunoblot analysis. Results from such analysis indicated the presence of Gα_13_ in Ric-8A-immunoprecipitates thereby demonstrating the interaction between endogenous Gα_13_ and ectopically expressed Ric-8A in SKOV3 cells (Figure 2A). In addition, we checked for the presence of other Gα-proteins in the Ric-8A immunoprecipitates. Gα_12_, the other member of the G12 family, as well as Gα_q_, which has been previously shown to interact with Ric-8A [21,22], could be observed in the HA-Ric-8A immunoprecipitates. In addition, Gα_11_, which is closely related to Gα_q_, was observed in the Ric-8A immune-complex. However, only low levels of Gα_i2_ could be detected in the HA-Ric-8A-immunoprecipitate. Since previous studies have shown an interaction between Ric-8A and Gα_i_ [13,17,18], the observed apparent “weaker” interaction between Gα_i2_ and Ric-8A in our studies could be due to the low level expression of Gα_i2_ in these cells or due to low avidity of the Gα_i2_-antibodies used in in the western blot analysis.

To examine the endogenous interaction between Gα_13_ and Ric-8A, we immunoprecipitated Gα_13,_ from SKOV3 or HeyA8 (2 × 10^6^ cells) ovarian cancer cell lysates and evaluated the co-precipitation of Ric-8A (Figure 2B). The Gα_13_-immunoprecipitates from the cell lysates indicated the presence of Ric-8A, thereby revealing the endogenous interaction between Gα_13_ and Ric-8A. Similarly, a reciprocal immunoprecipitation analysis using Ric-8A antibodies showed the presence of Gα_13_ in Ric-8A-immunoprecipitates in the lysates derived from both SKOV3 and HeyA8 cells (Figure 2C). Thus our results firmly establish the endogenous, *in vivo* interaction between Gα_13_ and Ric-8A.

### Ric-8A-interacting Domain of Gα_13_

**Figure 3:**
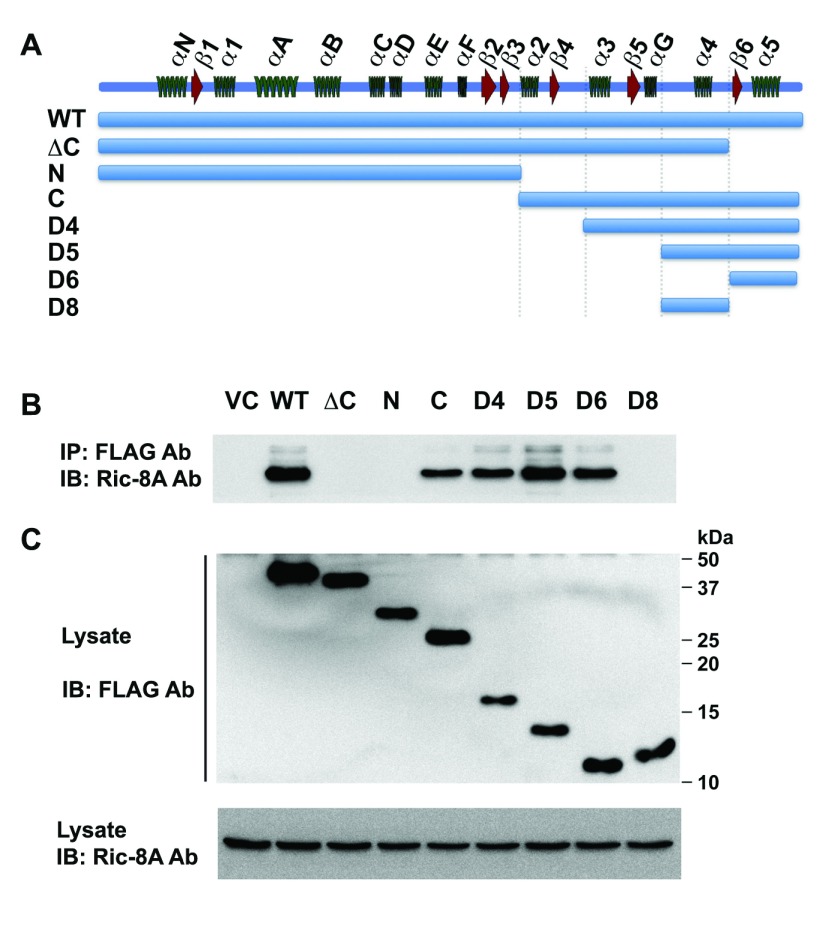
Mapping the Ric-8A-interacting domain of Gα_13_. **A.** Diagrammatic representation of the secondary structure of Gα_13_ and the respective deletion constructs of Gα_13_. All of the Gα_13_-constructs are FS-tagged at the N-terminal. **B.** Presence of Ric-8A was analyzed in the FLAG immunoprecipitation from cells transfected with vector control or the truncated constructs of Gα_13_. **C.** Expression levels of the truncated mutants of Flag-tagged Gα_13_ and Ric-8Awere monitored by immunoblot analysis of the lysates from the transfectants using antibodies to FLAG-epitope (*Upper Panel*) or Ric-8A (*Lower Panel*) respectively. The data presented are representative of three independent experiments with similar results.

It has been observed that ADP-ribosylation of Cys-341 of Gα_i1_ by pertussis toxin or the deletion of its C-terminus, more specifically the C-terminal 12 amino acids of Gα_i1,_ resulted in the loss of its interaction with Ric-8A [23,24,25]. To define whether Gα_13_ interacts with Ric-8A in a similar fashion, a series of deletion mutants of Gα_13_ (Figure 3A) were tested for their ability to interact with Ric-8A, by coimmunoprecipitation analysis. The deletion mutants of Gα_13_ (Figure 3A) were cloned into pcDNA3-FS vector and each of these Gα_13_-domain constructs was transiently expressed in HEK293 cells (2 × 10^6^ cells). 48 hours after transfection, the respective Gα_13_-domains were immunoprecipitated from the lysates using FLAG-antibodies. The presence of Ric-8A in the immunoprecipitates was monitored by immunoblot analysis using Ric-8A antibodies. Our results indicated that the minimal Gα_13_-domain required for its interaction with Ric-8A was the domain D6 that spans amino acids 342 to 377, which includes secondary structure b6 and a5 of the Gα-subunits [26]. While all of the N-terminal deletion constructs of Gα_13_ that contain this stretch of amino acids retained their ability to interact with Ric-8A, deletion of this domain resulted in the loss of interaction between Gα_13_ with Ric-8A (Figure 3B; Lane D5 versus D8). Thus, in addition to establishing that the interaction of Gα_13_ with Ric-8A is similar to Gα_i1_ in involving its C-terminus [23,24,25], our results reveal that the C-terminal b6-a5 alone is sufficient for Gα_13_ to interact with Ric-8A (Figure 3B).

### Effect of Ric-8A on Gα_13_-signaling

**Figure 4:**
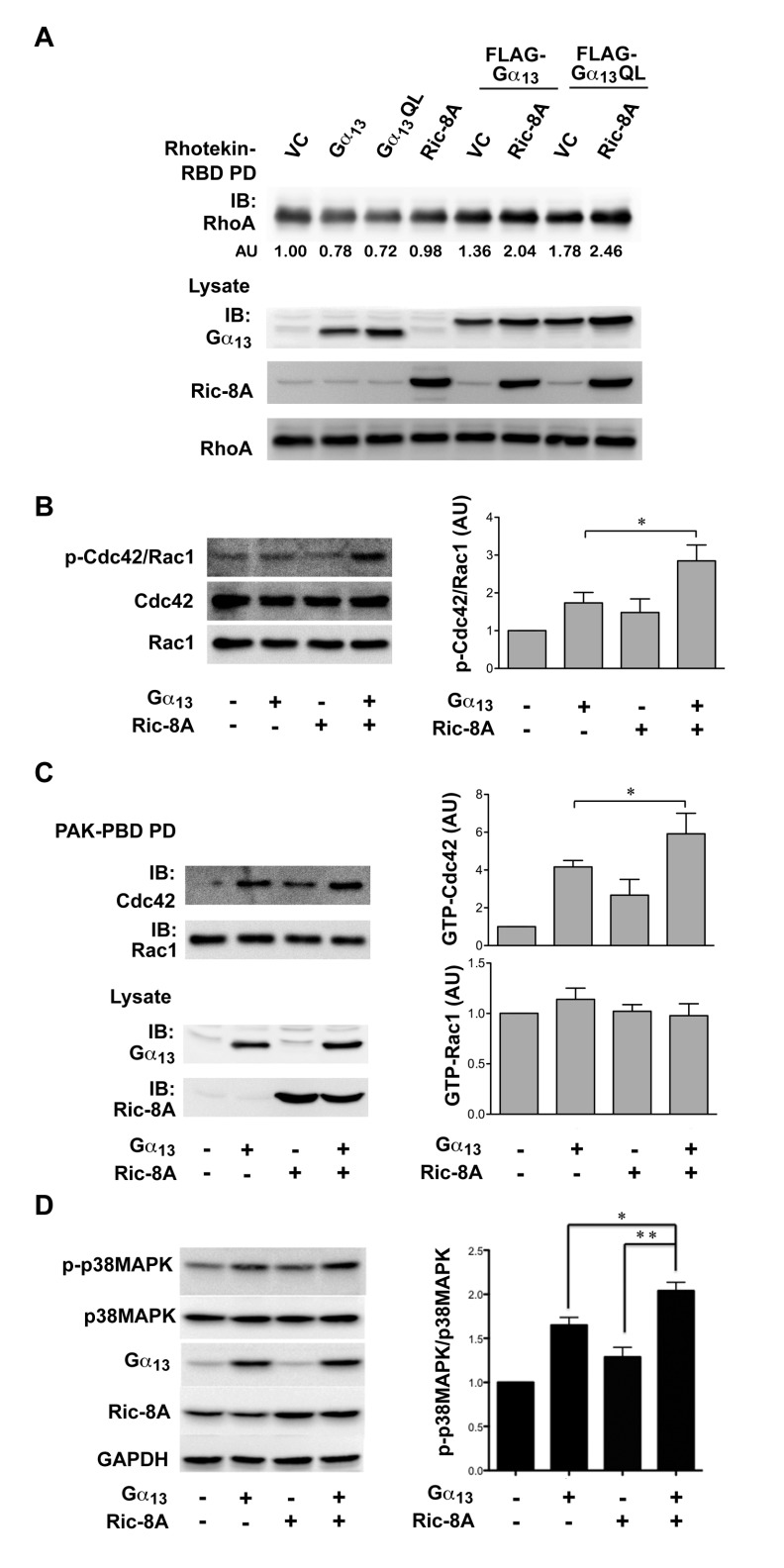
Ric-8A enhances Gα_13_-signaling Outputs. **A.** Activation of RhoA was analyzed based on the ability of Rho-binding domain (RBD) of Rhotekin to interact with activated RhoA. Vector control (VC) or constructs encoding Gα_13,_ Ric-8A, Gα_13_QL, Gα_13_ + Ric-8A or Gα_13_QL + Ric-8A were transiently expressed in HEK293 cells for 48 hrs. GTP-bound active form of Rho in the lysates from the transfectants was pulled down using GST-RBD beads (RBD PD) and visualized by immunoblot analysis. The experiment was repeated thrice and the results are from a typical experiment. Activated RhoA levels were quantified and presented as arbitrary units (AU) under the respective activated Rho lanes. **B.** Activation of CDC42/Rac1 was monitored using antibodies that recognize Ser-71 phosphorylated, activated forms of both Cdc42 and Rac1. Vector control (VC) or constructs encoding Gα_13,_ Ric-8A or Gα_13_ + Ric-8A were transiently expressed in HEK293 cells for 48 hrs. Lysates from the transfectants were subjected to immunoblot analysis using antibodies against Ser-71 phosphorylated CDC42/Rac1. The blot was stripped and re-probed for the expression levels of Rac1 and CDC42. Band intensity of Ser-71 phosphorylated Cdc42/Rac1 (p-Cdc42/Rac1) was normalized by the band intensity of Rac1 in the lysates. Relative p-Cdc42/Rac1 was calculated by comparing their expression levels in vector control (Right Panel). AU, arbitrary units; mean ± SD, n=3; * p < 0.05. **C.** Activation of Rac1 and Cdc42 were analyzed based on the ability of PAK interact with activated Rac or Cdc42. GTP-bound active form of Cdc42 or Rac1 was pulled down by GST-PAK3 PBD beads (PBD PD) and analyzed by Western blot. Band intensity of GTP-bound Cdc42 or Rac1 in PBD PD was normalized by the band intensity of Cdc42 or Rac1 in lysate, respectively (Right Panel). Relative levels were calculated by comparing to the level in vector control. (AU, arbitrary units; mean±SD, n=3; * p < 0.05). **D.** Activation of p38MAPK was monitored using antibodies specific for the phosphorylated activated form of p38MAPK. SKOV3-ip cells (1 × 10^6^) were transiently transfected with either vector control, Ga_13_, or Ric-8A constructs as indicated. At 48 hrs, the transfectants were lysed and the lysates were subjected to immunoblot analysis for phosphorylated p38MAPK using antibodies specific to phospho-p38MAPK. The blot was stripped and re-probed for the expression levels of p38MAPK and GAPDH to ensure equal loading. The stripped blots were also probed for the expression levels of Gα_13_ and Ric-8A using respective antibodies. The phosphorylated levels of p38MAPK in relation to total levels of p38MAPK were quantified and presented as bar graph in which the bar represent mean ± SD; n=3 (Right Panel). An unpaired two-tail *t*-test with Welch’s correction was performed to determine statistical significance, * p < 0.05.

It has been suggested that Ric-8A interaction with the Gα-subunits leads to the potentiation of signaling by the respective α-subunits. This has been attributed to the role of Ric-8A as a guanine nucleotide exchange factor [13,17], a molecular chaperone that facilitates the proper folding of Gα-subunit during their biosynthesis [27,28], or a factor that inhibits the ubiquitination of the α-subunits [29]. Therefore, we investigated whether the interaction between Gα_13_ and Ric-8A results in the potentiation Gα_13_-signaling. Since Gα_13_ is known to stimulate the Rho-family of GTPases, we tested whether the co-expression of Gα_13_ and Ric-8A would lead to an enhanced activation of any of these small GTPases. First, we investigated whether Ric-8A potentiates the activation of Rho by Gα_13_. SKOV3 cells were transfected with vectors encoding wild-type Gα_13_ with or without the coexpression of Ric-8A. At 48 hrs, the lysates derived from these cells were subjected to Rho pull-down assay using GST-fused Rho-binding domain of Rhotekin (GST-RBD). The Glutathione-Sepharose sequestered GST-RBD precipitate was analyzed for the presence of associated activated RhoA by immunoblot analysis. Results from such analyses indicated that the coexpression of Ric-8A along with Gα_13_ led to a small but consistent activation of Rho in these cells (Figure 4A). Next, we investigated whether Ric-8A potentiates Gα_13_-mediated activation of Rac1 and/or Cdc42. It has been demonstrated that Ser-71 phosphorylation of Rac1/CDC42 can be used to monitor the activation status of Rac1 and/or CDC42 [30,31]. While the initial studies suggested an inhibitory role for Ser-71 phosphorylation [32], recent studies have shown that Ser-71-phosphorylatioin is indicative of an activated and possibly an effector-specific state of Rac1/Cdc42 [33,34]. Therefore, we monitored Ser-71 phosphorylation status of Rac1/Cdc42. SKOV3 cells were transfected with vectors encoding wild-type Gα_13_, Ric-8A, or Gα_13_ plus Ric-8A, along with appropriate control. At 48 hrs, the lysates from these transfectants were analyzed for Ser-71 phosphorylated Rac1/CDC42 by immunoblot analysis using an antibody specific to phosphorylated Ser-71 of Rac1/Cdc42. As shown in Figure 4B, the coexpression of Ric-8A significantly increased the phosphorylation of Cdc42/Rac1. Since this assay does not distinguish between Rac1 and Cdc42, we also used pull-down assay using GST-fused P21-binding domain of PAK3 (GST-PAK-PBD) to identify whether Ric-8A-mediated potentiation of Gα_13_-signaling involves Rac1, Cdc42, or both. Lysates were prepared from SKOV3 cells in which wild-type Gα_13_ was transiently expressed for 48 hours along with or without Ric-8A. Activated Rac1 and CDC42 from the lysates were sequestered by GST-PAK-PBD and pulled down using GST-Sepharose beads. The presence of activated Rac1 or Cdc42 in the pulled down beads were monitored by immunoblot analysis using respective antibodies. Results from our analysis indicated that Ric-8A preferentially enhanced the Gα_13_-mediated activation of Cdc42 (Figure 4C). It has been shown previously that Gα_13_ specifically activates RhoA and Cdc42-specific events without affecting Rac-activation in some cellular contexts [35,36,37]. In addition, it has been observed that the stimulation of Cdc42 by Gα_13_ can lead to the activation of stress activated protein kinases and p38MAPK [36,38,39,40]. Therefore, we investigated whether Ric-8A enhances the activation of p38MAPK. SKOV3-ip cells were transfected with expression vectors encoding Gα_13_, Ric-8A, or Gα_13_ plus Ric-8A along with appropriate controls. 48 hours after transfections, the lysates from these cells were analyzed for the activation of p38MAPK by immunoblot analysis using antibodies specific to phosphorylated activated form of p38MAPK. Results from such analyses clearly indicated that the coexpression of Ric-8A potentiated Gα_13_-mediated activation of p38MAPK (Figure 4D). Taken together our results indicate that Ric-8A potentiates the Cdc42-p38MAPK signaling output from Gα_13_.

### Gα_13_-stimulated tyrosine-phosphorylation of Ric-8A

**Figure 5:**
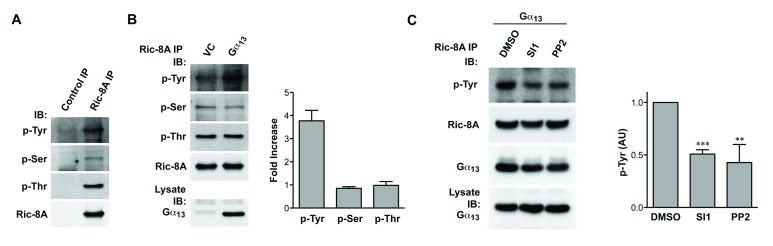
Gα_13_ induces Src-dependent tyrosine phosphorylation of Ric-8A. **A.** Lysates of HEK293 cells expressing Ric-8A was incubated with normal mouse IgG (Control IP) or anti-Ric-8A antibody and precipitated by protein-G resin. Immunoprecipitation was analyzed for the presence of tyrosine, serine and threonine phosphorylation (p-Tyr, p-Ser, p-Thr) of Ric-8A by immunoblot (IB). **B.** HEK293 cells were co-transfected with Ric-8A expressing construct along with vector control (VC) or Gα_13_ construct. Expression of Gα_13_ was confirmed in lysate. Immunoprecipitation of Ric-8A was analyzed for p-Tyr, p-Ser and p-Thr. Data are representative of three independent experiments with similar results. Band intensity of p-Tyr, p-Ser and p-Thr was normalized by the band intensity of Ric-8A, and the ratio of phosphorylation in the presence versus absence of Gα_13_ was calculated as fold increase (mean ± SD; n = 3). **C.** HEK293 cells transfected with Ric-8A and Gα_13_ were treated with vehicle control (DMSO) or 10 ΟM Src kinase inhibitor (SI1 or PP2) overnight. Ric-8A was immunoprecipitated and analyzed for p-Tyr. Immunoprecipitated Ric-8A was also analyzed for the presence of co-immunoprecipitated Gα_13_. In addition, Gα_13_ that was present in the lysates was also monitored. Results are representative of three independent experiments with similar results. Quantification of these results is presented in the Right Panel. Band intensity of p-Tyr was normalized by the band intensity of Ric-8A (AU, arbitrary units), and relative p-Tyr was calculated by comparing to the p-Tyr level in vehicle control. Mean ± SD, n=3; ** p < 0.01; *** p < 0.001.

Mass spectrometric analysis of Ric-8A from Gα_13_ using TAP approach, indicated that Ric-8A is phosphorylated at Tyr-435, Ser-436, Thr-441, and Thr-443 (Supplemental Figure S1). To confirm this, ectopically expressed Ric-8A was immunoprecipitated from HEK293 cells and the presence of phospho-tyrosine, phospho-serine and phospho-threonine was examined by immunoblot analysis, using antibodies specific to these phosphorylated residues. We observed the phosphorylation of Ric-8A at all of these residues (Figure 5A). More interestingly, co-expression of Gα_13_ led to a 4-fold increase in the level of tyrosine phosphorylation of Ric-8A without having such drastic effect on phospho-serine or phospho-threonine (Figure 5B). In this context, it is worth noting that Gα_13_ has been shown to activate Src-family of tyrosine kinases in many different cell types [41,42]. Therefore, we reasoned that the increase in tyrosine-phosphorylation of Ric-8A, observed with the expression of Gα_13,_ could be mediated by the Src-family of tyrosine kinases. To validate this reasoning, we tested two pharmacological inhibitors that target Src-family of tyrosine kinases. Experimentally, HEK293 cells transfected with Gα_13_, were treated with Src inhibitor 1 (SI1) or PP2 for 16 hrs, Ric-8A was immunoprecipitated, and the immunoprecipitates were subjected to immunoblot analysis using antibodies specific to phospho-tyrosine. Our results indicated that both SI1 and PP2 inhibited the tyrosine-phosphorylation of Ric-8A by 50% (Figure 5C), thereby suggesting a role for the Src-family of tyrosine kinases in Gα_13_-mediated Tyr-phosphorylation of Ric-8A. Results from this analysis also indicated that these inhibitors did not affect the interaction between Gα_13_ and Ric8A (Figure 5C).

### Gα_13_-induced, Src-dependent translocation of Ric-8A to plasma membrane

**Figure 6:**
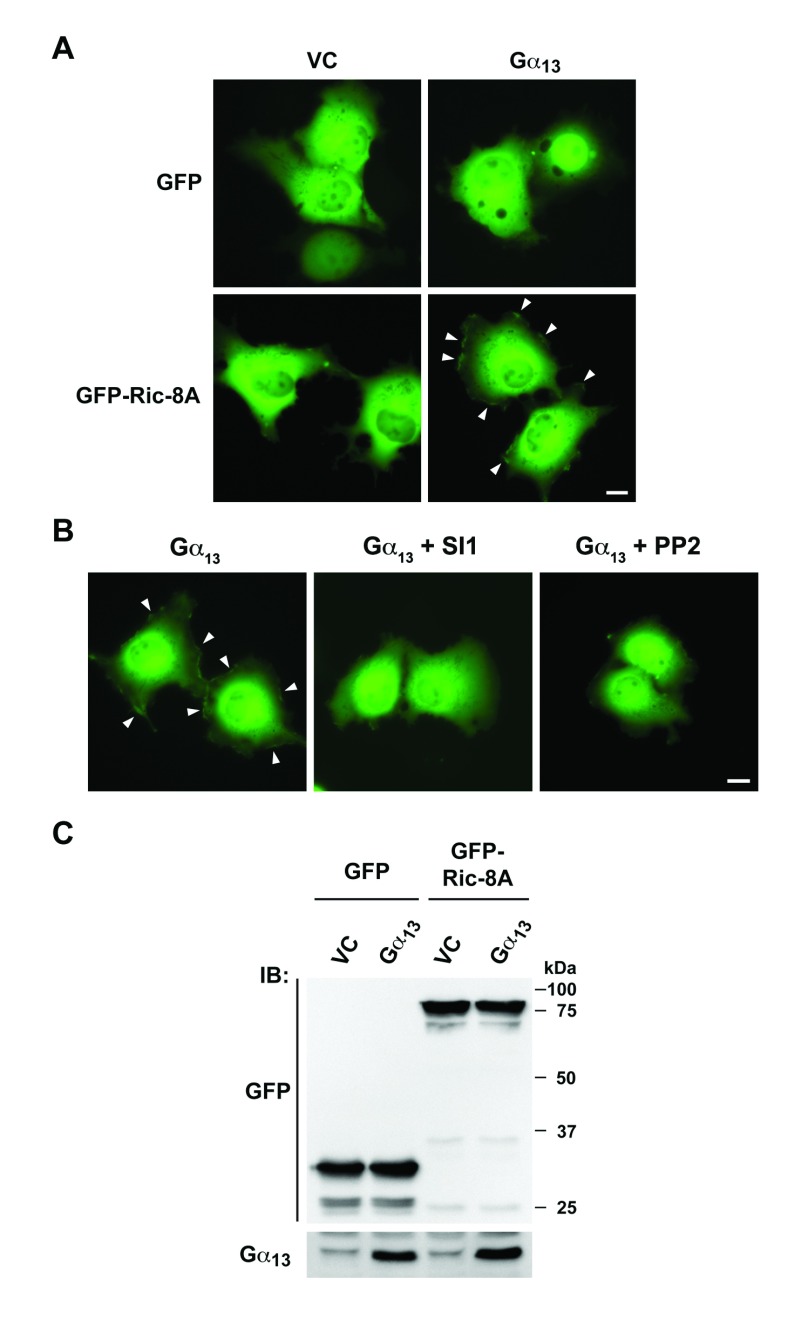
Gα_13_ induces Src-dependent plasma membrane translocation of Ric-8A. **A.** GFP or GFP-Ric-8A fusion construct was coexpressed with vectors expressing Gα_13_ along with appropriate control vectors (1 mg) in COS-7 cells (1.5 × 10^6^ cells/dish). At 48 hrs, the transfectants were lysed and the lysates were subjected to immunoblot (IB) analysis using the respective antibodies to monitor the expressions of GFP, GFP-Ric-8A and Gα_13_ proteins. **B.** Cells transiently expressing GFP or GFP-Ric-8A fusion protein along with vectors expressing Gα_13_ or vector control for 48 hrs were imaged by fluorescence microscopy. Localization of GFP-Ric-8A on plasma membrane (arrowheads) was observed in the presence of Gα_13_. Scale bar, 10 mm. **C.** COS7 cells transfected with GFP-Ric-8A and Gα_13_ for 48 hrs were treated 10mM Src kinase inhibitor SI1 or PP2 for 16 hrs and the fluorescence of GFP was imaged. Plasma membrane localization (arrowheads) was attenuated in the presence of SI1 or PP2. Scale bar, 10 mm. Data are representative of three independent experiments with similar results.

At least in the case of Gα_q_-signaling, it has been shown that Ric-8A is translocated to plasma membrane following the stimulation of Gα_q_-coupled receptor [21,22]. In the light of our results with Gα_13_-Ric-8A, we sought to investigate whether Gα_13_ is also capable of enacting plasma membrane translocation of Ric-8A. Therefore, we engineered an expression construct of Ric-8A in which GFP was fused to the N-terminus of Ric-8A. COS-7 cells were co-transfected with vectors encoding GFP or GFP-Ric-8A with or without vector encoding Gα_13_. The expressions of GFP, GFP-fused Ric-8A, and Gα_13_ were verified in the transfectants by immunoblot analysis using antibodies to GFP (Figure 6A). At 48 hours following transfection, the transfectants were imaged by fluorescence microscopy. As shown in figure 6B, similar to GFP-alone control, GFP-Ric-8A showed a diffused fluorescent signal in the cytoplasm, indicating that Ric-8A is predominantly localized in the cytosol (Figure 6B, Left Panel). However, when Gα_13_ was coexpressed with GFP-Ric-8A, the translocation of GFP-Ric-8A to cellular periphery could be observed (Figure 6B, Right Panel). This translocation was specific to Ric-8A since the coexpression of Gα_13_ with GFP-vector alone failed to elicit such translocation of GFP. These results demonstrate that Gα_13_ induces the translocation of Ric-8A to plasma membrane (Figure 6B). Furthermore, treating the transfectants that express GFP-Ric-8A and Gα_13_ along with the inhibitors targeting Src-family of kinases – SI1 or PP2 – potently attenuated Gα_13_-induced translocation of Ric-8A (Figure 6C), indicating that the tyrosine phosphorylation is a critical event for the translocation of Ric-8A.

## Discussion

It has been increasingly realized that the signaling outputs from G-proteins are fine-tuned at several stages of signal amplification, by mechanisms involving an array of signal modulators including, atypical GEFs, GDIs, GAPs, RGS proteins, AGS proteins, and ‘rheostat” proteins [43–46]. Ric-8A appears to be part of such a novel family of signal modulators [47]. Mammalian Ric-8A was initially identified as a Gα_i_-, Gα_o_-, Gα_q_-interacting proteins in a yeast two-hybrid screen [13,22]. Prior to our present study, *in vitro* studies using GST-Ric-8A-bound Glutathione-Sepharose beads, have shown a relatively poor/weak interaction between Ric-8A and Gα_13_ [13]. However, our results clearly demonstrate that Gα_13_ interacts with Ric-8A more robustly *in vivo* and there is a bidirectional functional modulation between these two proteins. In addition to validating the known interactions of Gα_13_ with Rho-GEFs and Ric-8A, the Gα_13_-based TAP analysis couple mass spectrometric analysis has identified Ric-8A as one of the major proteins that interact with Gα_13_ (Figure 1 & 2).

In many aspects, the interaction between Gα_13_ and Ric-8A is similar to the ones described for Gα_i1_. Similar to previous findings with Gα_i1_ [13,17], we also observed that Ric-8A interacts equally well with wild-type Gα_13_ as well as the constitutively active configurations Gα_13_QL (Figure 1D). Analogous to the observations with Gα_i1_ [23,24,25], our results indicate that the C-terminus of Gα_13_ is involved in its interaction with Ric-8A (Figure 3). However, our studies expand this further by demonstrating that the C-terminal domain of Gα_13_ containing b6-a5 domain can efficiently interact and coimmunoprecipitated with Ric-8A (Figure 3). Ric-8A has been demonstrated to be involved in promoting the dissociation of GDP from the Gα-subunits, thereby stabilizing a transient nucleotide-free configuration of the α-subunit to which GTP-loading can readily occur due to the relatively higher levels of GTP in the cytosol [13,17,18]. It is worth noting here that the previous structural studies have defined the C-terminal b6-a5 domain of Gα-subunits as the binding domain for the guanine ring of GDP/GTP [26]. Taken together with our findings that the b6-a5 domain of Gα_13_ is the critical domain involved in Ric-8A binding (Figure 3), it can be surmised that Ric-8A stimulates the release of GDP and stabilizes the nucleotide-free transition state of the α-subunit by directly interacting with the guanine-ring binding, b6-a5 domains of Gα_13_. Thus our results presented here, demonstrating the interaction of Ric-8A with the guanine-ring binding domain of Gα_13_, provide a structural basis for the functional role of Ric-8A. We are pursuing further studies to identify the critical amino acid residues within the b6-a5 domain of Gα_13_ involved in Ric8A interaction so that the inter-relationship between Ric8A binding site and the sites involved in of GDP/GTP binding and release could be defined.

Using ectopically expressed Ric-8A, we could demonstrate that Ric-8A interacts with the endogenous Gα_i2_, Gα_q_, Gα_11_, Gα_12_, and Gα_13_. More significantly, we could demonstrate and validate the *in vivo* interaction between endogenous Ric-8A and Gα_13_ (Figure 3). By using ovarian cancer cells in which the signaling by Gα_13_ is quite pronounced [15,20,48], we could entrap and demonstrate the robust endogenous interaction between Ric-8A and Gα_13_. There is accumulating evidence that Ric-8A is crucial for GPCR-mediated signaling. For example, Ric-8A has been shown to enhance LPA-stimulated ERK activation in Chinese hamster ovary cells [49]. It has also been observed that the activation of ERK by Gα_q_ was also affected by the knockdown of Ric-8A in 293T cells [22]. Our findings that Ric-8A potentiates the activation of Cdc42 -albeit RhoA - and downstream p38MAPK add further evidence to such signal-potentiation role for Ric-8A. The observation that the coexpression of Ric-8A with Gα_13_ potentiates the activation of RhoA and Cdc42, but not Rac1 (Figure 4), requires some clarification. Although the upstream signaling events as well as GEFs involved in the activation of Rac1 and Cdc42 overlap in many instances, these small GTPases are also known to be differentially regulated in a context-specific manner. A case to the point is that Gα_13_ specifically signals to RhoA or Cdc42 in several cellular and physiological contexts. While the mechanisms underlying such differential regulation of Rac1 and Cdc42 remain unclear, the observation that Ric-8A potentiates the activation of Cdc42 with little or no effect on Rac1 suggests the interesting possibility that Ric-8A could be involved in channeling Gα_13_-signaling to a specific effector(s). While it has been demonstrated that Ric-8A could act as a molecular chaperone in directing nascent Gα-subunits to plasma membrane [27,28], it appears that it is involved in facilitating functional specificity as well. While it is quite likely that the interaction of Ric-8A to specific α-subunit(s) is context- and/or cell-type-specific, it has been observed that in *Drosophila* the interaction of Ric-8A with Gα_i_ is involved mitotic spindle alignment [50,51,52] whereas Ric-8A interaction with Cta protein (a Drosophila homolog of G12/13) is involved in folded gastrulation pathway during embryonic development [53]. Further studies should define whether the mammalian Ric-8A is also involved in such spatiotemporal functional specificity.

Another interesting observation presented here relates to the phosphorylation of Ric-8A. One study has identified the phosphorylation of Ric-8A at Ser-502 during the early stages of mitosis as a part of “chromosomal passenger complex” in HeLa cells [54]. Several other serine, threonine, and tyrosine residues of Ric-8A that could be potentially phosphorylated were identified through large-scale quantitative phosphoproteomic analysis [55]. While these studies have identified the potential phosphorylation sites in Ric-8A, the mechanism through which they are phosphorylated or physiological contexts in which Ric-8A is phosphorylated, is not known. Our mass spectrometric analysis data of Ric-8A in the Gα_13_ tandem affinity purification shows that Ric-8A is phosphorylated at Tyr-435, Ser-436, Thr-441, and Thr-443 (Figure S1 A–C). Immunoblot analyses presented here further confirm that Ric-8A is phosphorylated at Ser-, Thr-, and Tyr-residues (Figure 5A). The observation that Gα_13_ potently and specifically stimulates the Tyr-phosphorylation of Ric-8A is quite novel and highly significant (Figure 5B). Based on the fact that these phosphorylatable residues are highly conserved across even distantly related species (Figure S2) it can be suggested that phosphorylation plays a critical role in regulating Ric-8A function. The findings that Src-inhibitors attenuate Gα_13_-mediated Tyr-phosphorylation of Ric-8A (Figure 5C), and the subsequent translocation of Ric-8A to membrane points, unravel the critical requirement of Tyr-phosphorylation in the membrane localization of Ric-8A. This is in line with the ability of Gα_13_ to stimulate the activity of Src [56,57]. The observation that the putative Src-family inhibitors failed to exert complete inhibition of the Tyr-phosphorylation of Ric-8A (Figure 4E & F) suggest the possibility that Gα_13_-mediated the Tyr-phosphorylation of Ric-8A could involve an alternative mechanism as well. Nevertheless, these potential mechanisms need not be mutually exclusive. While the mass spectrometric analysis has indicated the Tyr-435 as the Tyr-phosphorylation site of Gα_13_-TAP-purified Ric-8A (Figure S1), the precise downstream signaling events involved in this process needs to be defined. Likewise the kinases that are involved in the phosphorylation of Ser-436, Thr-441, and Thr-443 of Ric-8A and the underlying mechanisms involved in these phosphorylations remain to be elucidated. It is highly significant to note here that the Src-inhibitors do not impair the interaction between Gα_13_ and Ric-8A. Thus it appears that Src-activation is not required for Gα_13_-Ric-8A interaction and Gα_13_-Ric-8a interaction precedes Src-activation and subsequent phosphorylation of Ric-8A.

Previous studies have shown that Ric-8A translocates to plasma membrane in response to the activation of Gα_q_-coupled m1 muscarinic acetylcholine receptor when it binds with the ligand carbachol [21,22]. An interesting aspect of the tyrosine phosphorylation observed here is that the tyrosine phosphorylation of Ric-8A appears to be critical for the translocation of Ric-8A to plasma membrane (Figure 6). On the other hand, Ric-8A may also be important for the appropriate localization of heterotrimeric G proteins. In the absence of functional Ric-8, *Drosophila* Gα_i_ and its cognate Gb-subunits fail to be localized at the plasma membrane [50]. A recent report has also demonstrated that knockdown of Ric-8A prevented the translocation of Gα_13_ to the cell cortex in mouse embryonic fibroblasts [58]. Thus, it is possible that the observed Ric-8A and Gα_13_ regulate each other for proper spatial orientations required for efficient G protein signaling. We are in the process of defining the spatiotemporal and functional significance of the translocation of Ric-8A using the Gα_13_-expression model system presented here.

**Figure 7:**
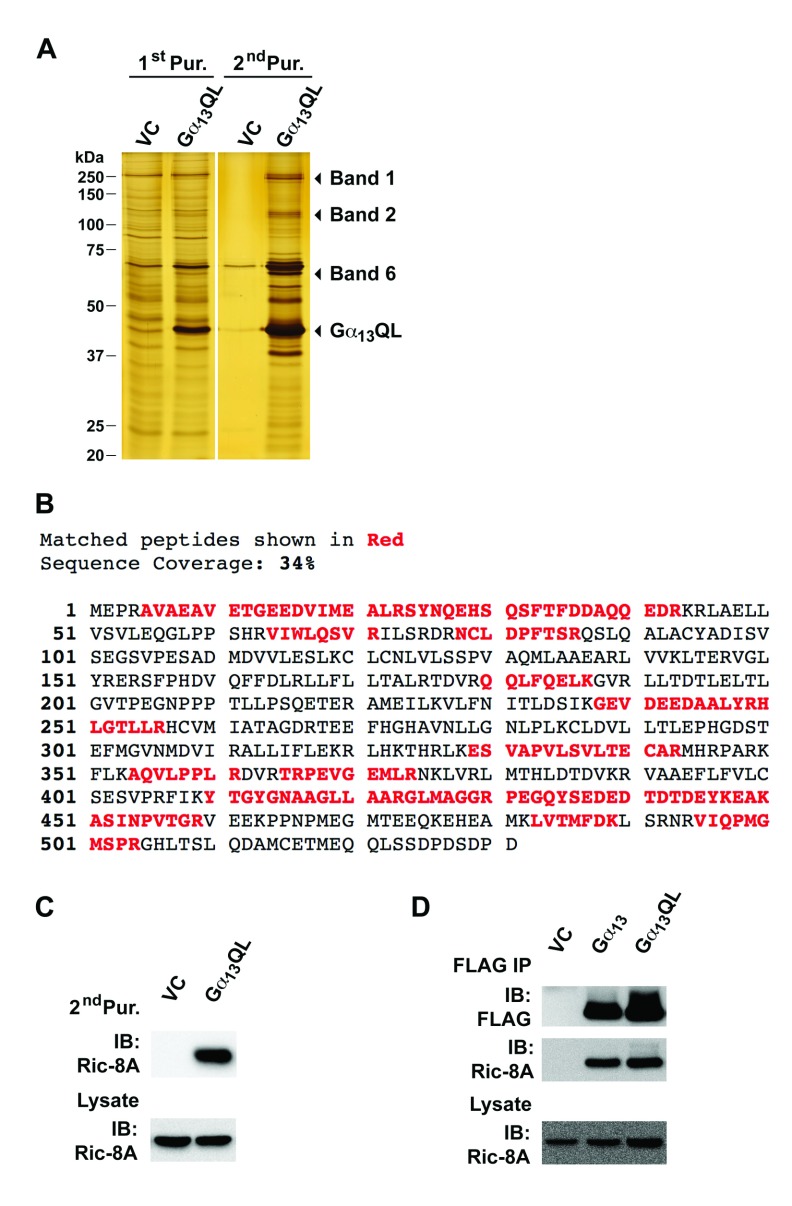
Signaling Paradigm involving Gα_13_-Ric-8A Interaction. Gα_13_ interacts with Ric-8A and stimulates the tyrosine phosphorylation of Ric-8A at Tyr-435 through a mechanism involving Src-family of tyrosine kinases. Gα_13_-mediated tyrosine phosphorylation plays an essential role in Gα_13_-mediated translocation of Ric-8A to plasma membrane events so as to promote the translocation. Ric-8A, in turn, enhances Gα_13_ signaling output such as the activation of RhoA, Cdc42, and downstream p38MAPK (see text for details).

Our results presented here, along with the previous findings, suggest a signaling paradigm in which nascent Gα_13_ stimulates Tyr-phosphorylation of Ric-8A and subsequent translocation of Ric-8A to plasma membrane through a mechanism involving Src-family of tyrosine kinases. In turn, Ric-8A potentiates Gα_13_-signaling, either as a non-canonical GEF that facilitates GTP-loading or a spatiotemporal signal coordinator that chaperones Gα_13_ to specific signaling response (Figure 7). Further studies should define the spatiotemporal sequence of events underlying this process.

## Supporting Information Available

PDF document containing Supplemental Figure S1 “Mascot Search Analysis of Gα_13_-TAP purified Ric-8A” and Supplemental Figure S2 “Conserved Phosphorylation Residues in Ric-8A”.

## Competing Interests

The authors declare that they have no competing interests.

## Appendix

### Abbreviations

Cdc42: Cell division control protein 42 homolog

Gα_12_ and Gα_13_: Heterotrimeric guanine nucleotide binding protein α subunits of the G12 family

Gα_q_ and Gα_11_: Heterotrimeric guanine nucleotide binding protein α subunits of the Gq family

GFP: Green fluorescent protein

GST: Glutathione S-transferase

HA: Hemagglutinin

LARG: Leukemia associated Rho guanine nucleotide exchange factor

MALDI-TOF: Matrix-assisted laser desorption/ionization–time-of-flight mass spectrometer

p115 RhoGEF: p115 Rho guanine nucleotide exchange factor

PAK3: p21 protein-activated kinase 3

PBD: p21 protein-binding domain

RBD: Rho-binding domain

Rac1: Ras-related C3 botulinum toxin substrate 1

Ric-8A: Resistance to inhibitors of cholinesterase 8 homolog A
