# Supplementary material for: Gα13 Stimulates the Tyrosine Phosphorylation of Ric-8A
Source: J Mol Signal. 2015 Jul 27;10:3. doi: 10.5334/1750-2187-10-3 (PMC4831272; doi:10.5334/1750-2187-10-3)
Supplement: Supplementary file 1 [file jms-10-00131-s001.pdf]

A.

Protein View

Match to: **RIC8A_HUMAN** Score: **245**
Synembryn-A OS=Homo sapiens GN=RIC8A PE=1 SV=3
Found in search of yan6-222.pkl

Nominal mass (M_r): **60185**; Calculated pI value: **5.20**
NCBI BLAST search of RIC8A_HUMAN against nr
Unformatted sequence string for pasting into other applications

Taxonomy: Homo sapiens

Fixed modifications: Carbamidomethyl (C)
Variable modifications: Acetyl (Protein N-term),Oxidation (M),Phospho (Y),Phospho (ST)
Cleavage by Trypsin: cuts C-term side of KR unless next residue is P
Sequence Coverage: **34%**

Matched peptides shown in **Bold Red**

```
  1 MEPRAVAEAV ETGEEDVIME ALRSYNQEHS QSFTFDDAQQ EDRKRLAELL
 51 VSVLEQGLPP SHRVIWLQSV RILSRDRNCL DPFTSRQSLQ ALACYADISV
101 SEGSVPESAD MDVVLESLKC LCNLVLSSPV AQMLAAEARL VVKLTERVGL
151 YRERSFPHDV QFFDLRLLFL LTALRTDVRQ QLFQELKGVR LLTDTLELTL
201 GVTPEGNPPP TLLPSQETER AMEILKVLFN ITLDSIKGEV DEEDAALYRH
251 LGTLLRHCVM IATAGDRTEE FHGHAVNLLG NLPLKCLDVL LTLEPHGDST
301 EFMGVNMDVI RALLIFLEKR LHKTHRLKES VAPVLSVLTE CARMHRPARK
351 FLKAQVLPPL RDVRTRPEVG EMLRNKLVRL MTHLDTDVKR VAAEFLFVLC
401 SESVPRFIKY TGYGNAAGLL AARGLMAGGR PEGQYSEDED TDTDEYKEAK
451 ASINPVTGRV EEKPPNPMEG MTEEQKEHEA MKLVTMFDKL SRNRVIQPMG
501 MSPRGHLTSL QDAMCETMEQ QLSSDPDSDP D
```

B.

Sort Peptides By ⦿Residue Number ○Increasing Mass ○Decreasing Mass

Start - End	Observed	Mr(expt)	Mr(calc)	Delta	Miss	Sequence	
5 - 23	1016.5593	2031.1040	2030.9830	0.1210	0	R.AVAEAVETGEEDVIMEALR.S	
5 - 23	1024.5555	2047.0964	2046.9779	0.1185	0	R.AVAEAVETGEEDVIMEALR.S	
24 - 43	811.3649	2431.0729	2430.9949	0.0780	0	R.SYNQEHSQSFTFDDAQQEDR.K	
64 - 71	500.8362	999.6578	999.5865	0.0713	0	R.VIWLQSVR.I	
78 - 86	555.2892	1108.5638	1108.4971	0.0667	0	R.NCLDPFTSR.Q	
180 - 187	517.3270	1032.6394	1032.5604	0.0791	0	R.QQLFQELK.G	
238 - 249	683.8423	1365.6700	1365.6048	0.0652	0	K.GEVDEEDAALYR.H	
250 - 256	405.2844	808.5542	808.4919	0.0623	0	R.HLGTLLR.H	
329 - 343	815.9901	1629.9656	1629.8396	0.1260	0	K.ESVAPVLSVLTECAR.M	
354 - 361	447.3160	892.6174	892.5494	0.0680	0	K.AQVLPPLR.D	
365 - 374	602.3571	1202.6996	1202.6077	0.0919	0	R.TRPEVGEMLR.N	
410 - 423	699.4310	1396.8474	1396.7099	0.1376	0	K.YTGYGNAAGLLAAR.G	
424 - 450	1051.1248	3150.3526	3150.2050	0.1476	1	R.GLMAGGRPEGQYSEDEDTDTDEYKEAK.A	2 Phospho (ST)
424 - 450	1056.4541	3166.3405	3166.1999	0.1406	1	R.GLMAGGRPEGQYSEDEDTDTDEYKEAK.A	Phospho (Y); Phospho (ST)
451 - 459	457.7925	913.5704	913.4981	0.0723	0	K.ASINPVTGR.V	
483 - 489	427.2628	852.5110	852.4415	0.0695	0	K.LVTMFDK.L	
483 - 489	435.2608	868.5070	868.4364	0.0706	0	K.LVTMFDK.L	
495 - 504	558.3201	1114.6256	1114.5627	0.0629	0	R.VIQPMGMSPR.G	
495 - 504	566.3333	1130.6520	1130.5576	0.0944	0	R.VIQPMGMSPR.G	
495 - 504	574.3123	1146.6100	1146.5526	0.0575	0	R.VIQPMGMSPR.G	

C.

Peptide View

Assigned Phosphorylation Sites

MS/MS Fragmentation of **GLMAGGRPEGQYSEDEDTDTDEYKEAK**
Found in **RIC8A_HUMAN** in **SwissProt**, Synembryn-A OS=Homo sapiens GN=RIC8A PE=1 SV=3
Match to Query 48: 3166.340472 from(1056.454100,3+) intensity(325.3111) index(11)

Monoisotopic mass of neutral peptide Mr(calc): 3166.1999
Fixed modifications: Carbamidomethyl (C) (apply to specified residues or termini only)
Variable modifications:
M3 : Oxidation (M), with neutral losses 0.0000(shown in table), 63.9983
Y12 : Phospho (Y) — **Tyr 435**
T18 : Phospho (ST), with neutral losses 97.9769(shown in table), 0.0000 — **Thr 441**
Ions Score: 24 **Expect:** 2.8
Matches : 57/692 fragment ions using 128 most intense peaks

MS/MS Fragmentation of **GLMAGGRPEGQYSEDEDTDTDEYKEAK**
Found in **RIC8A_HUMAN** in **SwissProt**, Synembryn-A OS=Homo sapiens GN=RIC8A PE=1 SV=3

Match to Query 47: 3150.352572 from(1051.124800,3+) intensity(327.6839) index(21)

Monoisotopic mass of neutral peptide Mr(calc): 3150.2050
Fixed modifications: Carbamidomethyl (C) (apply to specified residues or termini only)
Variable modifications:
S13 : Phospho (ST), with neutral losses 97.9769(shown in table), 0.0000 — **Ser 436**
T20 : Phospho (ST), with neutral losses 97.9769(shown in table), 0.0000 — **Thr 443**
Ions Score: 9 **Expect:** 97
Matches : 23/476 fragment ions using 62 most intense peaks

Supplemental Figure S1. Mascot Search Analysis of Gα_{13}-TAP Purified Ric-8A. The MS/MS dat was analyzed using the Mascot MS/MS Ion Serach (version 2.2, Matrix Science, Boston, MA) with the following parameters: Carbamidomethylation of cysteine residues was specified a a fixed modification.Acetylation of protein N-terminus, oxidation of methionine residues, and phosphorylation of serine, threonine, and tyrosine residues were allowed as variable modifications. The mass values were monoisotopic and the protein mass was unrestricted. Peptide mass tolerance and fragment mass tolerance were 1.2 and 0.6 Daltons, respectively. Two missed cleavages were allowed with trypsin.
Panel A shows the matched peotides (red) in the full length Ric-8A sequence (amino acid residues 1-531).
Panel B shows the list of analyzed peptides and the matched phosphopeptides.
Panel C shows the peptide analyses that assign the phosphorylated residues to Tyr 435, Ser 436, Thr 441, and Thr 443.
