# Supplementary material for: Gα13 Stimulates the Tyrosine Phosphorylation of Ric-8A
Source: J Mol Signal. 2015 Jul 27;10:3. doi: 10.5334/1750-2187-10-3 (PMC4831272; doi:10.5334/1750-2187-10-3)
Supplement: Supplementary file 2 [file jms-10-00131-s002.pdf]

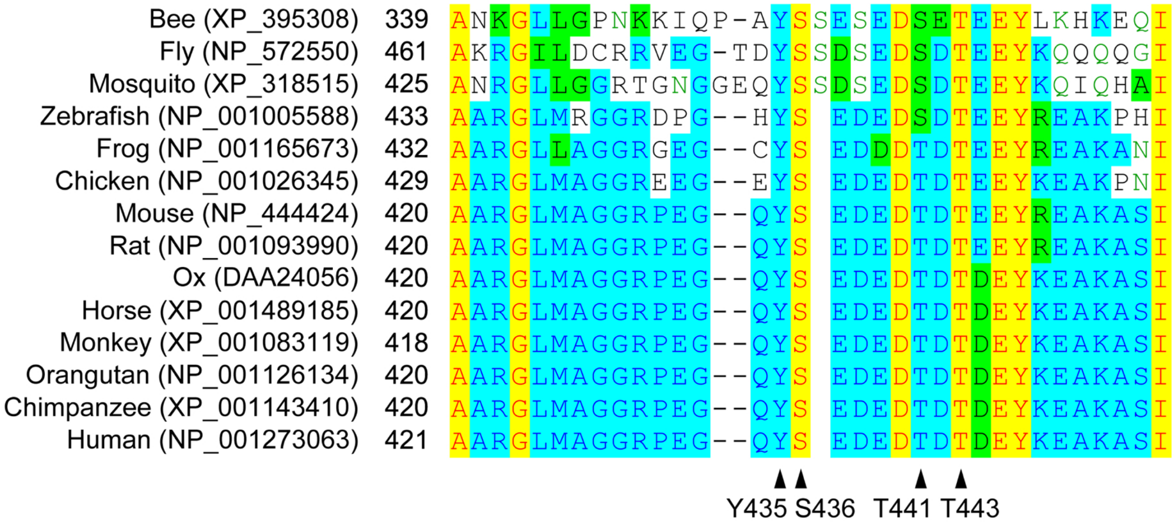

Supplemental Figure S2. Conserved Phosphorylation Residues of Ric-8A. Multiple sequence alignment analysis was carried out using a modified Clustal W algorithm on the AlignX module of Vector NTI software (Invitrogen, Carlsbad, CA). Residue-alignments are color-coded as follows:

Red font on yellow block: Fully conserved

Blue font on cyan block: Similar amino acids

Black font on green block: Conserved at greater than 50%

Green font: Weak similarity
